# Virulence studies of *Enterobacter sakazakii *isolates associated with a neonatal intensive care unit outbreak

**DOI:** 10.1186/1471-2180-8-64

**Published:** 2008-04-18

**Authors:** Stacy Townsend, Edward Hurrell, Stephen Forsythe

**Affiliations:** 1 School of Science and Technology, Nottingham Trent University, Clifton Lane, Nottingham, NG11 8NS, UK

## Abstract

**Background:**

In 1994, an outbreak of *Enterobacter sakazakii *infections in France occurred in a neonatal intensive care unit during which 17 neonates were infected. More than half of the infected neonates had severe clinical symptoms; 7 cases of necrotising enterocolitis (one with abdominal perforation), one case of septicemia, and one case of meningitis. The other 8 neonates were shown to be colonized but remained asymptomatic. There were three deaths. Four distinguishable pulsotypes of *E. sakazakii *were isolated during the outbreak, and the deaths were attributable to one pulsotype. This paper compares strains, from the four pulsotypes, for attachment and invasion of mammalian intestinal cells, macrophage survival and blood-brain barrier invasion. A fourth death from septic shock also occurred during the *E. sakazakii *outbreak. This was due to *E. cloacae *which at the time of the outbreak had been misidentified as *E. sakazakii*. This isolate has been included in this study.

**Results:**

All *E. sakazakii *strains attached and invaded Caco-2 human epithelial cells, and invaded rat brain capillary endothelial cells. The majority of strains persisted in macrophage cells for 48 h. Two strains from fatal NEC and meningitis cases showed the highest invasion rate of Caco-2 intestinal cells. Their invasion of brain capillary endothelial cells was equivalent or greater than that of the neonatal *E. coli *meningitis strain K1. These strains also had extended spectrum β-lactamase activities. *E. cloacae *differed from *E. sakazakii *due to the greater attachment and less invasion of epithelial cells, no survival in macrophages, and less invasion of capillary endothelial brain cells.

**Conclusion:**

While variables such as host factors and treatment strategies determine the outcome of infection, our *in vitro *studies evaluated the virulence of the isolates associated with this outbreak. It was not possible to directly correlate clinical symptoms and outcomes with *in vitro *studies. Nevertheless, we have shown the variation in invasive potential of *E. sakazakii *with intestinal and blood-brain barrier cells between and within pulsotypes from a neonatal intensive care unit outbreak. *E. sakazakii *strains were able to persist and even replicate for a period within macrophage cells. These traits appear to facilitate host immune evasion and dissemination.

## Background

*Enterobacter sakazakii *is an opportunistic pathogen associated with the ingestion of reconstituted infant formula, and is a rare cause of neonatal meningitis, necrotising enterocolitis (NEC) and sepsis [1-3]. These cases often occur among low-birth weight preterm neonates, who are generally more susceptible to Gram-negative bacterial sepsis and endotoxemia which is associated with NEC [4,5]. The bacterium can persist under desiccated conditions in infant formula for over two years [6]. There have been a number of reported *E. sakazakii *outbreaks which have been attributed to reconstituted infant formula which may be contaminated at source or during preparation [7-11]. The virulence of *E. sakazakii *has been studied by Pagotto *et al. *[12] and Mange *et al. *[13] who showed the presence of enterotoxins and adhesion to brain cells, respectively. The influence of endotoxin on increased translocation of intestinal bacteria and *E. sakazakii *in the neonatal rat was demonstrated by Townsend *et al*. [14]. Recently, Townsend *et al*. [15] comparatively investigated several *E. sakazakii *strains showing chronic-patterned brain inflammation in neonatal rats following intracranial inoculation. These studies further demonstrated the ability of *E. sakazakii *to invade brain capillary endothelial cells and persist in human macrophages. The IL-10/IL-12 ratio secreted by these macrophages was high and suggests a type 2 immune response may be activated early during *E. sakazakii *infection that is inadequate to clear the infection [15]. Previously, we reported a NICU outbreak in which 17 neonates were infected, and 3 neonates died [6]. Three distinguishable *E. sakazakii *pulsotypes were isolated from neonates and reconstituted formula. A fourth pulsotype was isolated from an unopened tin of powdered infant formula. Pulsotype 2 was associated with the three deaths, and therefore was potentially more virulent. A pulsotype is determined by pulsed field electrophoresis (PFGE) and refers to strains which are not distinguishable by treatment with rare cutting DNA restriction enzymes. However, some strains in pulsotype 2 possessed extended spectrum beta-lactamase activities, and therefore some genetic variability existed between these strains. A summary of the *E. sakazakii *pulsotypes and neonates is given in Table 1. Previous publications have not reported the individual case details associated with the isolates under study. Therefore, it is not possible to directly consider correlations between *in vitro *and *in vivo *studies. This is the first report studying *in vitro *virulence assays using clinical isolates with associated patient details, symptoms and outcomes.

**Table 1:** Description of *E. sakazakii *and *E. cloacae *strains used in this study

Organism	Strain number	Neonate	Isolation site	Symptoms	Isolation date (day/mn/yr)	PFGE profile
*E. sakazakii*	696	D	Stools	NEC II^b^	08/06/1994	1
*E. sakazakii*	701	F	Peritoneal fluid	NEC III^b ^(died)	07/04/1994	2
*E. sakazakii*	767^a^	H	Trachea	Meningitis (died)	11/05/1994	2
*E. sakazakii*	709	C	Trachea	Septicaemia	12/05/1994	2
*E. sakazakii*	695^a^	J	Trachea	NEC II (died)	07/06/1994	2
*E. sakazakii*	693	Q	Stools	Asymptomatic	18/06/1994	3
*E. sakazakii*	716		Infant formula		11/07/1994	4
*E. cloacae*	766	R	Blood culture	Sepsis (died)	04/06/1994	NA^c^

a Strains demonstrated extended spectrum β-lactamase activities.

b NEC Stage II (definite NEC) is characterised by the systemic signs of temperature instability, apnoea, bradycardia, lethargy with additional symptoms of absence of bowel sounds, radiological signs of intestinal dilation, ileus and pneumatosis intestinalis. NEC Stage III (advanced NEC) is where the neonate is critically ill with metabolic acidosis, neutropenia, signs of generalised peritonitis, marked tenderness and distension of abdomen.

c NA, Not appropriate

## Results

### *In vitro *virulence determination

**Figure 1:**
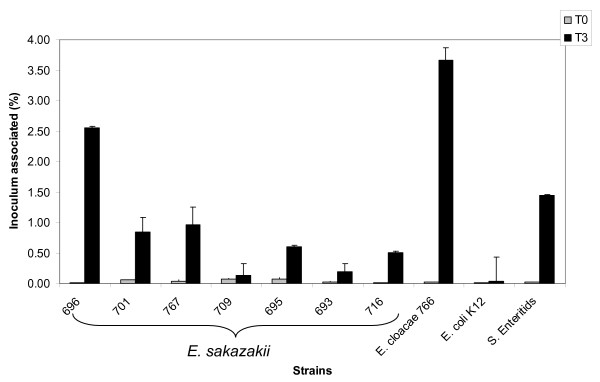
Bacteria attached to the Caco-2 epithelial cell surface was enumerated after 3 h by subtracting the number of bacteria recovered following gentamicin protection assay at the same time interval, as described in Fig 2. Results are presented as the percentage of the inoculum associated. Data are means ± standard errors of two independent experiments performed in triplicate. *E. coli *K12 and *S*. Enteritidis were used as negative and positive controls, respectively.

The attachment and invasion of Caco-2 human epithelial cells was determined after a 3-h exposure period with strains from each *E. sakazakii *pulsotype and the *E. cloacae *strain 766 from the outbreak. *S. *Enteritidis, a well documented enteric pathogen capable of attaching and invading Caco-2 cells was used as a positive control. Pulsotype 1 (strain 696) attached (2.6 ± 0.24% of inoculum) more than other pulsotypes and *S. *Enteritidis, (1.4 ± 0.24%), which was the positive control (Fig. 1). Pulsotype 2 strains (695, 701, 709 and 767) associated less with epithelial cells (0.96–0.14% of inoculum) than *S. *Enteritidis. Strains from pulsotypes 3 (693) and 4 (716) also attached, at relatively low values (0.19 and 0.50%, respectively) compared with *S. *Enteritidis but greater than the *E. coli *K12 negative control (0.04 ± 0.02%). *E. cloacae *strain 766 attached to Caco-2 cells more abundantly (3.7% of inoculum) than *S. *Enteritidis and the *E. sakazakii *strains. All strains showed significantly more attachment than *E. coli *K12 (p ≤ 0.001) and significantly more or less than *S*. Enteritidis (p ≤ 0.013).

**Figure 2:**
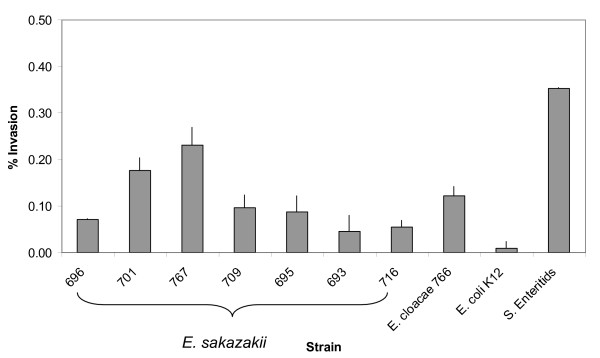
Bacterial invasion of Caco-2 epithelial cells was determined by gentamicin protection assay at 0 (T0) and 3 (T3) h. Results are presented as the percentage of the inoculum intracellular (% Invasion). Data are means ± standard errors of two independent experiments performed in triplicate. *E. coli *K12 and *S*. Enteritidis were used as negative and positive controls, respectively.

All strains invaded Caco-2 cells at a significantly (p ≤ 0.001) greater value than *E. coli *K12 (0.01% of inoculum), and less than *S. *Enteritidis (0.35% of inoculum)(Fig. 2). Strains 701 and 767 invaded at higher values, 0.17 and 0.23% of inoculum (p ≤ 0.02), respectively than the other *E. sakazakii *strains (0.04–0.12% of inoculum). While not statistically different, there did appear to be an apparent difference between strains 767 and 701 with *S. *Enteritidis.

**Figure 3:**
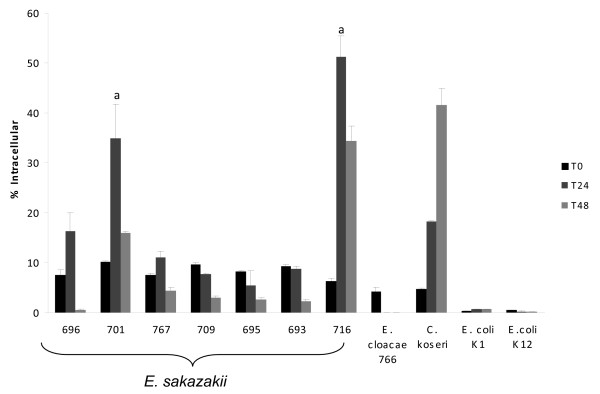
Human U937 macrophages were used in a gentamicin protection assay with mid-log phase bacteria. Intracellular bacteria were enumerated after 24 h (T24) and 48 h (T48). Results are presented as percent intracellular of the inoculum. Data are means ± standard errors of two independent experiments performed in triplicate. *E. coli *K1 (persists), *E. coli *K12 (killed), and *C. koseri *strain 319 (replicates) were used as controls.

After 24 h within U937 macrophages, significantly higher numbers of intracellular *E. sakazakii *were recovered from strains 701, a pulsotype 2 (p ≤ 0.04) from a fatal NEC case and 716, a pulsotype 4 isolated from powdered infant formula (p ≤ 0.001), than other *E. sakazakii *strains (Fig. 3). All *E. sakazakii *strains persisted and were recovered from macrophages at detectable levels at 48 h. In contrast to the *E. sakazakii *strains, there was significantly less uptake and persistence of *E. cloacae *strain 766 in U937 macrophages. At 24 h, the number of *E. cloacae *recovered was significantly less (p ≤ 0.05) than the *E. sakazakii *strains and was not detectable after 48 h.

**Figure 4:**
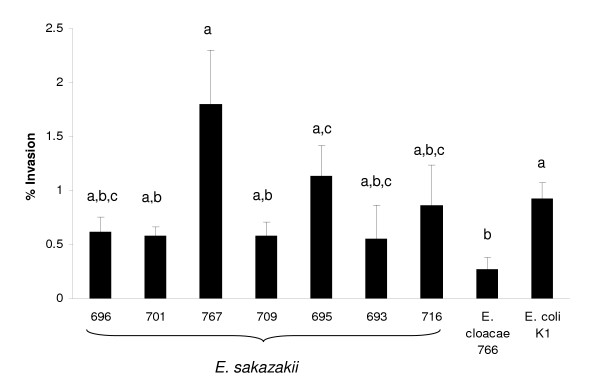
Gentamicin protection assay with rat rBCEC4 cells. Bacteria were grown overnight, inoculated and incubated for 1.5 h with rBCEC4 cells then treated with gentamicin for 30 min. *E. coli *K12 (non-invasive) and *E. coli *K1 (invasive) were used as controls. No bacteria were recovered at T0 following gentamicin treatment or following inoculation with *E. coli *K12. Results for T2 are presented as percent intracellular of the initial inoculation after 2 h. Strains without significant difference from *E. coli *K1 (p ≤ 0.18), 716(p ≤ 0.06) and 695(p ≤ 0.10), are designated by letter (a-c), respectively. All experimental strains had p values not significantly different from invasive (marked 'a'). Data are means ± standard errors from two independent assays performed in triplicate.

*E. sakazakii *is associated with rare cases of bacterial meningitis. Therefore, we carried out an *in vitro *study to investigate the ability of *E. sakazakii *to invade capillary endothelial cells which comprise the blood-brain barrier. *E. sakazakii *strains from the 4 pulsotypes were used for invasion studies of rat brain capillary endothelial cells (rBCEC4). Invasion was evaluated after inoculation (1.5 h) and gentamicin treatment (0.5 h). The *E. coli *K12 negative control did not invade this cell line (detection limit 50 cfu/1 × 10^5 ^rBCEC4 cells, data not shown). However, *E. sakazakii *strain 767 invaded rBCEC4 cells significantly more than *E. sakazakii *strains 693, 696, 701, 709 and *E. cloacae *strain 766 (p < 0.043; Fig. 4). Strain 767 was from the fatal meningitis case (neonate F). *E. sakazakii *strain 695 invaded rBCEC4 cells significantly more than *E. sakazakii *strains 701, 709 and *E. cloacae *strain 766 (p < 0.017; Fig. 4). Thus, on average, strains 767, 695, and 716 were the most invasive. Furthermore, all strains tested (except *E. coli *K12 negative control and *E. cloacae *strain 766) had p values (p ≤ 0.081) not significantly different from invasive meningitic *E. coli *K1 positive control.

## Discussion

The strains in this study were from the largest reported outbreak of *E. sakazakii *in a NICU, with the most deaths, that has been reported to-date [6]. In addition, it is the first report comparing *in vitro *virulence assays of clinical isolates with associated patient details and clinical symptoms.

*E. sakazakii *is associated with NEC, bacteraemia, sepsis and meningitis. Therefore, attachment to intestinal cells and translocation to systemic tissue are important in the pathogenesis of the organism. Variations in symptoms for each pulsotype could have varied according to dose, neonate age and antibiotic administration. However, pulsotype 2 appeared to be more virulent than pulsotypes 1 and 3 based on the number of NEC, septicaemia, meningitis and fatal cases. In addition, strains 695 and 767 in pulsotype 2 acquired extended spectrum β-lactamase activity [6]. It is accepted that other unidentified factors could have contributed to the number of neonatal cases and severity of infection, such as duration of exposure.

The attachment of *E. sakazakii *strains to Caco-2 cells after a 3-h exposure period was greater than the negative control (p ≤ 0.001) *E. coli *K12 (Fig. 1). Strain 696 (pulsotype 1) attached to a greater extent than the positive control *S. *Enteritidis (2.5 and 1.5% respectively). Strain 693 (pulsotype 3) was from an asymptomatic case and showed lower attachment values than the *E. sakazakii *strains which had caused NEC, and meningitis. The attachment of strain 716, from powdered infant formula, was significantly (p = 0.026) higher than 693 and 709 (p = 0.017) and similar to 695, 701 and 767. *E. cloacae *strain 766 showed the greatest attachment value (3.6%). There was no direct correlation between attachment and invasion rates of Caco-2 cells. For example, strains 696 and 766 had the highest attachment rates, yet 701 and 767 had the highest invasion rates (Fig. 1 &2). All *E. sakazakii *strains and *E. cloacae *showed similar potential for invasion. Strains 701 and 767, associated with fatal cases of NEC and meningitis, showed the highest invasion rates (0.18 and 0.23%). This suggests that the ability to invade intestinal cells may be a strong indication of potential virulence and poor outcome in the neonatal host.

Most *E. sakazakii *strains persisted in macrophages for 48 h. Strains 701 (pulsotype 2, fatal NEC case) and 716 (pulsotype 4 from powdered infant formula) multiplied significantly in the first 24 h. Persistence and replication within macrophages suggests that *E. sakazakii *possess virulence mechanisms to withstand the bactericidal activities within macrophages and evade the host immune response. Controls for macrophage persistence (*E. coli *K1), killing (*E. coli *K12), and replication (*C. koseri *SMT319) comparatively showed that *E. sakazakii *strains could persist within macrophages for at least 48 h and some strains demonstrated moderate (767 and 696) to high (701 and 716) levels of replication. In contrast to the *E. sakazakii *strains, there was either no uptake of *E. cloacae *(strain 766) by U937 macrophage cells, or it was rapidly killed following macrophage uptake.

*E. sakazakii *is associated with rare cases of bacterial meningitis. Therefore, the ability to invade cells comprising the blood-brain barrier was investigated using rat brain capillary endothelial cell line rBCEC4. *E. coli *K1 and *C. freundii *have been tested in both Human Brain Microvascular Endothelial Cells (HBMEC) and rBCEC4 cells and show similar levels of invasion, supporting the use of rBCEC4 cells as a model of BBB cell invasion [15]. The *E. sakazakii *strains and *E. cloacae *766 were able to invade rat brain cells at similar levels as *E. coli *K1 (Fig. 4). *E. coli *K1 is known to cause acute bacterial meningitis in neonates and further demonstrates the virulence potential of *E. sakazakii *for this host site. *E. sakazakii *is invasive for capillary endothelial cells and may gain access to the brain via crossing the normally impenetrable blood-brain barrier with specific virulence mechanisms [15].

The four pulsotypes have been previously described with respect to source, patient symptoms, and phenotypic traits [6]. Pulsotype 4 strains were isolated from an unopened tin of powdered infant formula and were not associated with any neonatal infections. It is notable that pulsotype 4 strains showed no protease activity on skimmed milk agar plates or capsule production. Whether these phenotypes are related to virulence determinants is unknown, but are under current investigation.

The outcome of infection due to *E. sakazakii *will be dependent upon a number of factors such as birth weight, age, immune status and antibiotic therapy [7]. In our study, it was not possible to directly correlate clinical symptoms and outcomes with *in vitro *studies. Nevertheless, we have shown the invasive potential of *E. sakazakii *with intestinal and blood-brain barrier cells. In addition, *E. sakazakii *was able to persist and even replicate for a period within macrophage cells. These traits appear to facilitate host immune evasion and dissemination. In addition, *E. sakazakii *can acquire antibiotic resistance during infection, as shown by the acquisition of extended spectrum β-lactamase activities by strains 695 and 767. These two strains were isolated from two neonates, one month apart (Table 1). There was variation within pulsotype 2 for attachment and invasion of intestinal and blood-brain barrier cells. Whether this reflected the variation in patient symptoms is uncertain. But it is notable that strain 767 had the highest level of endothelial cell invasion, greater than *E. coli *K1, and was isolated from a fatal meningitis case, the outcome of which could have been influenced detrimentally by the extended β-lactamase spectrum activity the strain had acquired due to its resistance to standard antibiotic therapy.

Strain 766 was initially identified as *E. sakazakii *and was isolated from a fatal case of septic shock during the period of the NICU *E. sakazakii *outbreak (Table 1). However, recent 16S rRNA gene sequencing revealed it was *E. cloacae *[6]. This strain showed attachment, and invasion of Caco-2 endothelial cells, no invasion or no survival in macrophages, and low invasion of rat brain capillary endothelial cells. Therefore, it had the ability to attach and invade intestinal cells, but no propensity for macrophage survival or dissemination, nor an ability to invade the brain. It had however been able to enter to bloodstream and cause fatal septic shock.

The potential attachment and invasion of host tissue, dissemination and invasion of the blood-brain barrier have been investigated and demonstrated the potential of *E. *11 *sakazakii *to infect exposed neonates. The source of the three pulsotypes could not definitely be attributed to contaminated reconstituted infant formula. Nevertheless the feeding practices of preparation for 24-h periods, and prolonged (>3 h) administration could have enabled bacterial growth in the reconstituted formula and increased the risk of infection.

## Conclusion

While variables such as host factors and treatment strategies determine the outcome of infection, our *in vitro *studies evaluated the virulence of the isolates associated with this outbreak. It was not possible to directly correlate clinical symptoms and outcomes with *in vitro *studies. Nevertheless, we have shown the variation in invasive potential of *E. sakazakii *with intestinal and blood-brain barrier cells between and within pulsotypes from a neonatal intensive care unit outbreak. *E. sakazakii *strains were able to persist and even replicate for a period within macrophage cells. These traits appear to facilitate host immune evasion and dissemination.

## Methods

### Bacterial strains

All *E. sakazakii *and *E. cloacae *strains were from the previously described outbreak in 1994. The source neonates are not identifiable and ethical committeee approval was not required for their reporting here [6] (Table 1). Non-pathogenic *Escherichia coli *K12 HB101, *E. coli *K1 neonatal meningitic CSF isolate RS218 (O18 : K1 : H7), *Citrobacter koseri *SMT319 (isolated from the cerebrospinal fluid of an infant with *C. koseri *meningitis and a brain abscess obtained from Joseph St Geme III, Washington University School of Medicine, USA), and *Salmonella enterica *serovar Enteritidis (NCTC 3046) from Nottingham Trent University culture collection, were included as control organisms for the *in vitro *virulence assays, as appropriate.

### Caco-2 attachment and invasion assays

The attachment and invasion assays were performed with strains of *E. sakazakii *and *E. cloacae *as given in Table 1. *E. coli *K12 and *S. *Enteritidis were negative and positive controls, respectively. The assays were primarily based on the method of Szymanski *et al*. [16].

#### Attachment assay

Mammalian cells were seeded at 2 × 10^4 ^per well overnight. The test organism was grown overnight on TSA at 37°C and harvested in tissue culture growth medium. The organism was then added at 2 × 10^6 ^cfu per well and incubated with 5% CO_2 _at 37°C for 3 h before washing (x3 in PBS), dislodging (trypsin-EDTA treatment) followed by lysis with Triton X-100. Bacterial counts were carried out on TSA at 37°C. Mammalian cell integrity following infection was qualitatively assessed using trypan blue staining after a 3-h incubation period. The control for bacterial attachment/invasion was obtained by immediately (<3 min) aspirating the bacteria off the mammalian cells after addition, prior to dislodging, lysis and plating on TSA. This assay enumerated the total number of bacteria associated with the mammalian cells after a 3 h incubation period. These may be attached to the surface or intracellular. Therefore, the number of attached bacteria was determined by subtracting the number of intracellular bacteria following invasion using the gentamicin treatment described below. Data are presented as the average number of bacterial colony forming units/well, given that each well contained 2 × 10^4 ^mammalian cells. Values were compared using the t-test (p = 0.05).

#### Invasion assays

As per bacterial attachment studies, mammalian cells were seeded at 2 × 10^4 ^per well and incubated overnight with 5% CO_2 _at 37°C. The test bacterial strain was grown overnight on TSA at 37°C and harvested in tissue culture growth medium. The bacterium was then added at 2 × 10^6 ^cfu per well and incubated with 5% CO_2 _at 37°C for 3 h. Afterwards, gentamicin was added (25 μg/ml) to each well and the cells were further incubated for 1 h before washing, dislodging, lysis and plating on TSA for viable counting. Mammalian cell integrity following infection was qualitatively assessed using trypan blue staining after the 3-h incubation period. Control counts were obtained by adding gentamicin (25 μg/ml) to each well and incubating for 1 h (reported as T0) prior to washing the mammalian cells, dislodging, lysis and plating on TSA.

Each strain was assayed in at least 2 independent assays. Each assay was performed in triplicate, and the bacterial viability was determined in duplicate. Selected strains have been assayed over a 6-month period to determine the experimental and biological variability of the assays. The level of detection was 100 cfu/well. Values were statistically assessed using the t-test (p = 0.05).

### U937 macrophage uptake studies

The macrophage uptake studies were performed with strains of *E. sakazakii*, *E. coli *K12 (negative control), *E. coli *K1 (positive control) and *C. koseri *(positive control). The method was based on that by [17] and as utilized in similar studies [15,18]. U937 macrophages were obtained from ATCC (CRL-1593.2) and seeded into 75 ml tissue culture flasks [19]. Cells were cultivated in RPMI 1640 medium with 2 mM L-glutamine, and modified to contain 10 mM HEPES, 1 mM sodium pyruvate, 4.5 g/L glucose, 1.5 g/L sodium bicarbonate, and supplemented with 10% foetal bovine serum. At least 24 h prior to infection, cells were treated with 0.1 μg/ml of phorbol 12-myristate 13-acetate (PMA; Sigma), placed in tissue culture plates and left at 37°C, under 5% CO_2 _to adhere and become activated. Cells were gently washed with RPMI to remove residual PMA, and fresh media was added prior to infection. U937 human macrophages were seeded at 10^6 ^cells per well. The bacteria were inoculated at 10^5 ^cfu per well for 45 min at 37°C in 5% CO_2_. After the incubation period, macrophages were re-suspended in U937 medium supplemented with 100 μg/ml of gentamicin and incubated for an additional 45 min at 37°C in 5% CO_2_. Macrophages were then washed twice, lysed with 0.5% Triton X100, serially-diluted and plated to determine the number of intracellular bacteria. Trypan blue exclusion staining indicated that macrophage viability was maintained. Results are presented as invasion efficiency expressed as the percent (%) of inoculum that is intracellular. Data are presented as the average number of bacterial colony forming units/well, given that each well contains 2 × 10^4 ^mammalian cells.

### Gentamicin protection assay of blood-brain barrier invasion model

Invasion assays were performed using rat brain capillary endothelial cell line 4 (rBCEC4), a kind gift from I. E. Blasig (Berlin, Germany). Cells were seeded at 1 × 10^5^/well into collagen coated 24-well plates and left to adhere for 48 h in media described by Blasig *et al. *[20]. Each bacterial strain was grown in BHI broth overnight, washed with PBS and inoculated in triplicate at an m.o.i. of 1:100. Inoculated cells were incubated with 5% CO_2 _at 37°C for 1.5 h as described by Badger *et al. *[21]. In order to quantify bacterial invasion, gentamicin was added (100 μg/ml) to each well and incubated for 30 min. Then the cells were washed twice with PBS, trypsin treated, lysed and dilutions were plated on NA. Cell integrity following invasion was qualitatively assessed using trypan blue staining after a 2-h incubation period. *E. coli *K12 (non-invasive) and *E. coli *K1 (invasive) were used as negative and positive controls. Data is presented as the percent invasion as determined by [100 × (number of bacteria recovered/number of bacteria inoculated)]. Values were statistically evaluated using the t-test.

## Authors' contributions

ST and EH designed and carried out the experiments, and analysed the data. SF was the principal investigator and supervised the project. SF drafted the manuscript. All authors revised the manuscript, and approved the final manuscript.
